# Vibro-Thermal Wave Radar: Application of Barker Coded Amplitude Modulation for Enhanced Low-Power Vibrothermographic Inspection of Composites

**DOI:** 10.3390/ma14092436

**Published:** 2021-05-07

**Authors:** Saeid Hedayatrasa, Joost Segers, Gaétan Poelman, Wim Van Paepegem, Mathias Kersemans

**Affiliations:** 1 Mechanics of Materials and Structures (UGent-MMS), Department of Materials, Textiles and Chemical Engineering (MaTCh), Ghent University, Technologiepark-Zwijnaarde 46, 9052 Zwijnaarde, Belgium; saeid.hedayatrasa@ugent.be (S.H.); joost.segers@ugent.be (J.S.); gaetan.poelman@ugent.be (G.P.); wim.vanpaepegem@ugent.be (W.V.P.); 2 SIM Program M3 DETECT-IV, Technologiepark-Zwijnaarde 48, 9052 Zwijnaarde, Belgium

**Keywords:** vibro-thermal wave radar (VTWR), vibrothermography, local defect resonance (LDR), barely visible impact damage (BVID), carbon fiber reinforced polymer (CFRP)

## Abstract

This paper proposes an efficient non-destructive testing technique for composite materials. The proposed vibro-thermal wave radar (VTWR) technique couples the thermal wave radar imaging approach to low-power vibrothermography. The VTWR is implemented by means of a binary phase modulation of the vibrational excitation, using a 5 bit Barker coded waveform, followed by matched filtering of the thermal response. A 1D analytical formulation framework demonstrates the high depth resolvability and increased sensitivity of the VTWR. The obtained results reveal that the proposed VTWR technique outperforms the widely used classical lock-in vibrothermography. Furthermore, the VTWR technique is experimentally demonstrated on a 5.5 mm thick carbon fiber reinforced polymer coupon with barely visible impact damage. A local defect resonance frequency of a backside delamination is selected as the vibrational carrier frequency. This allows for implementing VTWR in the low-power regime (input power < 1 W). It is experimentally shown that the Barker coded amplitude modulation and the resultant pulse compression efficiency lead to an increased probing depth, and can fully resolve the deep backside delamination.

## 1. Introduction

Active infrared thermography is a cost-effective non-destructive testing technique which enables fast full-field inspection of relatively large objects using a highly sensitive infrared camera [1,2]. The test-piece is generally excited with an external heat source so that a heat flow is stimulated throughout the sample and the defects are detected based on their impact on the thermal response recorded at the inspection surface. A surface heat flux can be induced by irradiating the exterior of the test-piece using, e.g., optical lamps or laser (optical thermography). The heat diffuses throughout the material and the thermal diffusivity mismatch at defect interfaces provide a means to detect defects. This needs a double “travelling” distance of the heat wave to the defect’s depth and back to the surface (while experiencing a highly damped 3D heat diffusion), which makes relatively deep defects hardly detectable. In fact, the thermal signature of the defect must be sufficiently high so that it dominates the non-uniform heating induced by the excitation source and the corresponding in-plane heat diffusion. Especially for composites with high in-plane diffusivity, the latter is of high concern. Proper post-processing of the thermographic dataset is essential in order to ensure maximum detectability [3,4,5,6]. The test-piece may also be inspected in the transmission mode such that the defects are detected based on the thermal response transmitted to the back surface, which leads to increased detectability of deep defects [7]. However, this approach requires access to both sides of the test-piece which limits its application for in situ non-destructive testing (NDT) and health monitoring of structural components. Anyhow, a defect may still be inaccessible due to its poor interaction with the stimulated heat wave, e.g., a closed crack with small effective disbond area or a crack oriented parallel to the heat flow.

Vibrothermography (also known as sonic thermography or thermosonics) is another active infrared thermography technique in which the test-piece is subjected to an external vibrational excitation, e.g., using an actuator bonded to the surface [8]. The dynamic response of the test-piece leads to activation of hardly detectable defects and makes them act as internal heating sources [9,10,11]. The vibration-induced heat generated at the defected area directly diffuses to the generally cold inspection surface and reveals the defect when its thermal signature is above the noise level of the infrared camera (i.e., 20 mK for a high-end cooled camera). However, adequate vibrational activation of the defects to a detectable limit generally requires very high excitation power of up to a few kilowatts [12].

The vibrational response, and so the heating efficiency at the defect, can be amplified by tuning the excitation at a resonance frequency of the test-piece [13,14]. In case of testing polymeric materials, this will further lead to efficient self-heating of the test-piece due to viscoelastic damping which reveals defects as areas with distinctive variation of self-heating (so-called self-heating based vibrothermography) [15].

Another approach is to tune the excitation frequency band at a local defect resonance (LDR) frequency, which enables low-power vibrothermography using a piezoelectric (PZT) wafer or an air-coupled transducer [16,17,18,19,20]. As LDR frequencies should be known a priori for the LDR based low-power vibrothermography, a more recent study by the current authors [21] has paved the way for a stand-alone identification of LDR frequencies through an efficient vibrothermographic spectroscopy procedure.

Sinusoidal amplitude modulation (AM) of the heating excitation for a number of cycles, so called lock-in thermography [22], increases the signal-to-noise ratio (SNR). Moreover, the probing depth can be tuned by the AM frequency which controls the diffusion length of the heat wave. In other words, the lower the frequency, the higher the diffusion length and as such the deeper the probing depth. Hence, the probing depth of lock-in thermography is limited due to its fixed AM frequency.

A frequency modulated excitation was initially implemented for broadband thermal wave imaging with optical excitation by Mandelis [23]. Furthermore, it was extended to the concept of thermal wave radar (TWR) [24,25,26] by adapting the pulse compression technique which was originally developed for increasing the range resolution and the SNR of radio wave radar systems. In TWR, a modulated waveform is used as the excitation signal and its cross-correlation with the corresponding thermal response is calculated. In this way, the impulse response of the sample (considered as a linear and time invariant system) to a pulsed excitation (Dirac delta-like stimulus) can be estimated, but with a higher SNR. In fact, the cross-correlation process compresses the energy of the signal under a main lobe whose peak value determines the strength of the reflected echo, while the associated delay time (or lag value) indicates the depth of the reflector. Analogue frequency modulated (sweep) and discrete phase modulated (Barker coded) excitations are the two widely researched types of modulated waveforms in TWR [25,27,28,29,30,31,32,33]. Recently, the current authors introduced a discrete frequency-phase modulated waveform which was the outcome of an optimization study. This novel frequency-phase modulated waveform outperforms the existing waveforms in terms of depth resolvability [34,35,36]. The TWR approach is not exclusive to the case of optical heating, and it has already been applied for enhanced performance of eddy current infrared thermography [37,38,39]. In vibrothermography, the concept of sinusoidal amplitude modulation is extensively studied, e.g., [16,20,40,41], resulting in lock-in vibrothermography (LVT). Application of TWR in vibrothermography has also been studied by Liu, et al. [42] through linear frequency modulation of the vibrational amplitude for high-power inspection of a metallic test coupon with flat-bottom holes. It was shown that the peak value of the compressed pulse has a higher SNR compared to the phase images obtained through LVT at different frequencies.

In this paper, a low-power Vibro-Thermal Wave Radar (VTWR) technique for inspection of composites is introduced. The VTWR procedure employs a vibrational excitation at a LDR frequency, which is modulated using a 5 bit Barker code, followed by application of the matched filter process. The peak, lag and phase of the compressed pulse is extracted and analyzed. The 5 bit Barker coded VTWR is compared to a 5 cycle LVT of the same AM frequency (i.e., the same excitation energy), and its outperformance in detection of very deep damage features is demonstrated.

The paper is organized as follows. In Section 2, the background of VTWR is provided and its enhanced depth resolvability is substantiated based on a 1D analytical model. In Section 3, experimental validation is provided for a 5.5 mm thick carbon fiber reinforced polymer (CFRP) coupon with barely visible impact damage (BVID). Various AM frequencies are tested and the deeper probing depth of VTWR is explicitly confirmed. Section 4 formulates the conclusions.

## 2. Vibro-Thermal Wave Radar (VTWR)

Depending on the gap or contact pressure of the defect’s interfaces and their morphology, different heating mechanisms are activated [9,43,44,45]. Among the various mechanisms, rubbing friction and viscoelastic damping predominantly contribute to the vibration-induced heating. Obviously, the frictional heating is exclusively activated in a defected area. However, the viscoelastic damping (or self-heating) is more significantly present at the areas with a higher strain energy density. This may be at a defected area due to local defect resonance [20,21], but also at a non-defected area due to global resonance of the test-piece [15,46,47]. In this section, a simplified 1D analytical model is used for simulating the surface thermal response in case of (vibration-induced) subsurface heating of a material with 5 mm thickness, using MATLAB (R2020a, MathWorks, Natick, MA, USA). VTWR is applied by binary phase modulation (5 bit Barker code) of the heat flux at the defect’s depth, and its depth resolvability is compared with LVT.

### 2.1. Thermal Frequency Response to Subsurface Heat Sources

**Figure 1:**
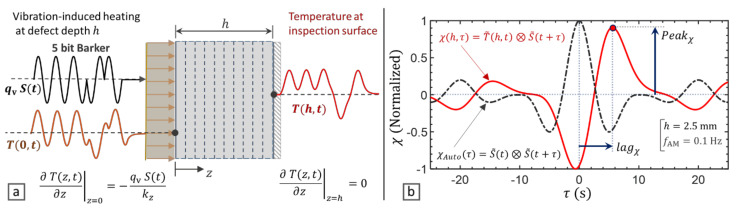
Analysis of the thermal response of a solid medium to subsurface heat sources and implementation of Vibro-Thermal Wave Radar (VTWR) using a 5 bit Barker coded waveform, (**a**) schematic model with heat dissipation-free boundary conditions, (**b**) VTWR for a 2.5 mm deep defect in a carbon fiber reinforced polymer (CFRP) material.

As schematically shown in Figure 1a, a heat source (i.e., defect) is modelled at a depth h of a solid medium. A uniformly distributed heat flux $q_{v}$ is applied which is modulated by a Barker coded signal $S\left(t\right)$.

In the absence of internal heating sources and lateral heat dissipation, the thermal response of the solid medium along the depth (*z*-axis) is governed by the 1D parabolic equation of heat diffusion [48]:

(1) $$\frac{\partial^{2}T\left(z,t\right)}{\partial z^{2}}-\frac{1}{\alpha_{z}}\frac{\partial T\left(z,t\right)}{\partial t}=0$$

(2) $$\alpha_{z}=\frac{k_{z}}{\rho C_{p}}$$

where T is temperature [K], t is time [s], $\alpha_{z}$ is thermal diffusivity [m^2^/s], $k_{z}$ is thermal conductivity [W/m K], ρ is the density [kg/m^3^] and $C_{p}$ is the heat capacity at constant pressure [J/kg K]. The vibration-induced heat is generated at the defect’s depth (i.e., z=0) and the thermal response of the inspection surface (i.e., z=h) is calculated by applying the heat dissipation-free boundary conditions given in Figure 1a.

By substituting the harmonic solution $T\left(z,t\right)=\theta \left(z,\omega \right)\exp\left(i\omega t\right)$ in Equation (1) and solving the differential equation, the steady-state thermal response to a mono-frequency excitation is derived as [34,49]:

(3) $$\theta \left(z,\omega \right)=\frac{q\left(\omega \right)}{\beta \left(\omega \right)k_{z}}\left(\frac{\exp\left(\beta \left(\omega \right)(z-2h\right)+\exp\left(-\beta \left(\omega \right)z\right)}{1-\exp\left(-2\beta \left(\omega \right)h\right)}\right)$$

where $i=\sqrt{-1}$, ω=2πf is the angular frequency [rad/s], $\beta \left(\omega \right)=\sqrt{i\omega /\alpha_{z}}$. Here, $q\left(\omega \right)$ is the frequency-domain heating load which, for a given heating amplitude $q_{v}$ and modulation signal $S\left(t\right)$, is calculated by:

(4) $$q\left(\omega \right)=F\left\{q_{v}S\left(t\right)\right\}$$

The time-domain thermal response of the inspection surface (z=h) is then derived from:

(5) $$T\left(h,t\right)=F^{-1}\left\{\theta \left(h,\omega \right)\right\}$$

where F and $F^{-1}$, respectively, denote Fourier and inverse Fourier transform operator.

### 2.2. Matched Filtering of the Vibro-Thermal Response

VTWR is implemented by matched filtering (i.e., cross-correlation) of the surface thermal response $T\left(h,t\right)$ with the modulation signal $S\left(t\right)$, as follows [50]:

(6) $$\chi \left(h,\tau \right)=\tilde{T}\left(h,t\right)⊗\tilde{S}\left(t+\tau \right)=\int_{-\infty }^{+\infty }\tilde{T}\left(h,t\right)\tilde{S}\left(t+\tau \right)dt$$

where ⊗ denotes cross-correlation and (˜) denotes the alternating (AC) component of the signal due to the mono-polar (heating only) nature of vibration-induced heating. In the analytical simulation, a purely harmonic (i.e., bi-polar, or heating-cooling) excitation is applied (i.e., $\tilde{S}=S$ and $\tilde{T}=T$). However, in practice this AC component is usually estimated by removing a low-order polynomial interpolant of the thermal response as the direct (DC) component [28,34,51]. For computational efficiency, the analysis is performed in the frequency domain as follows [50]:

(7) $$\chi \left(h,\tau \right)=F^{-1}\left\{\theta \left(h,\omega \right)\varsigma^{*}\left(\omega \right)\right\}$$

(8) $$\varsigma \left(\omega \right)=F\left\{W\left(t\right)\tilde{S}\left(t\right)\right\}$$

where the superscript (*) denotes the complex conjugate and W is a windowing function used for reducing the side lobes of the cross-correlation. In this study, a Hanning window is applied.

Due to the pulse compression efficiency of the Barker coded excitation signal, the cross-correlation $\chi \left(h,\tau \right)$ compresses the energy of the whole signal under a main peak as shown in Figure 1b, which its time delay (lag) corresponds to the depth of defect. The asymmetry and the lag introduced by the thermal response from a subsurface defect is readily illustrated in Figure 1b by comparison of $\chi \left(h,\tau \right)$ with the auto-correlation of the excitation signal $\chi_{Auto}\left(\tau \right)$ which is a sinc-like function. The peak value $Peak_{\chi }$ and corresponding lag $lag_{\chi }$ of the cross-correlation are then derived as:

(9) $$Peak_{\chi }=\mathrm{Max}\left(\chi \left(h,\tau \right)\right)$$

(10) $$lag_{\chi }={\left.\tau \right|}_{\chi \left(h,\tau \right)=Peak_{\chi }}$$

Subsequently, the phase of cross-correlation $\varphi_{\chi }$ can be found as:

(11) $$\varphi_{\chi }={\left.\tan^{-1}\left(\frac{\chi \left(h,\tau \right)}{\chi_{H}\left(h,\tau \right)}\right)\right|}_{\tau =0}$$

where $\chi_{H}$ represents the cross-correlation of $\tilde{T}\left(h,t\right)$ with the Hilbert transform of $\tilde{S}\left(t\right)$ [26].

In the case of mono-frequency harmonic excitation, the peak $Peak_{\chi }$ and the phase $\varphi_{\chi }$ of cross-correlation reduce to the well-known magnitude and phase of lock-in thermography. In this case, calculation of $lag_{\chi }$ is impractical due to the poor pulse compression quality.

### 2.3. Performance of Vibro-Thermal Wave Radar (VTWR)

**Figure 2:**
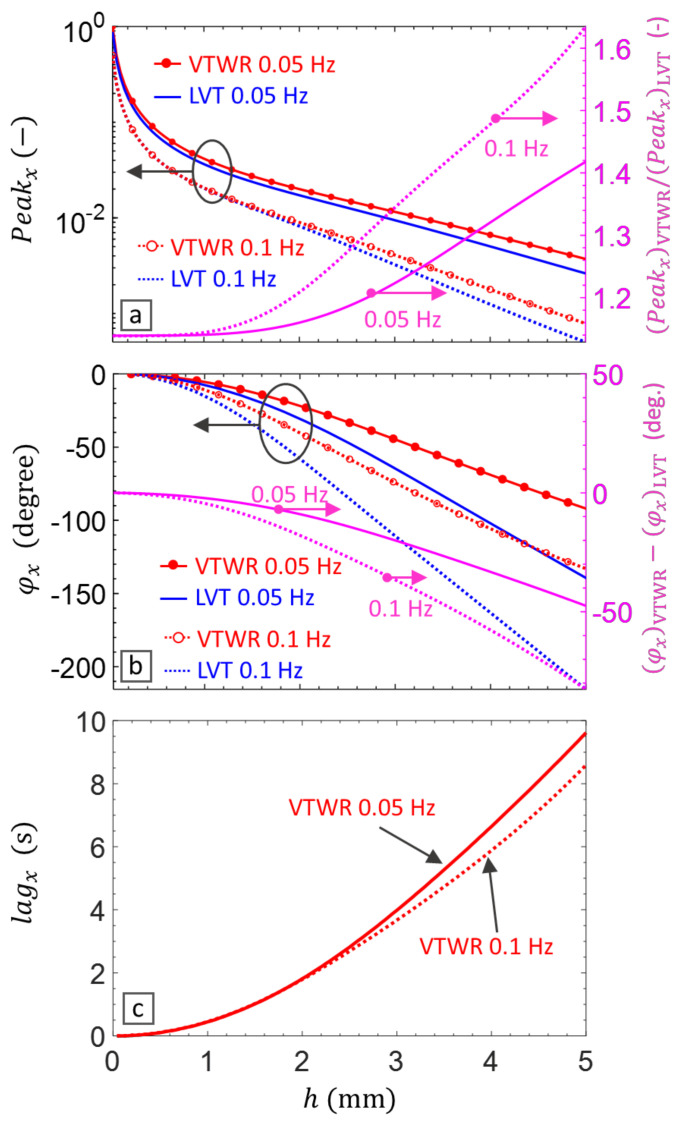
Simulation of VTWR using a 5 bit Barker coded waveform (see Figure 1a) at two AM frequencies 0.05 and 0.1 Hz for inspection of a 5 mm thick CFRP, and its comparison with 5 cycles LVT at the same AM frequency. (**a**) $Peak_{\chi }$, (**b**) $\varphi_{\chi }$ , and (**c**) $lag_{\chi }$.

In this section, VTWR is applied using a broadband 5 bits Barker coded waveform [50] as shown in Figure 1a. CFRP material with through-the-thickness thermal conductivity $k_{z}=0.53\;W/m\;K$, density $\rho =1530\;\mathrm{kg}/m^{3}$ and specific heat capacity $C_{p}=917\;J/\mathrm{kg}\;K$ [52] is modelled. The $Peak_{\chi }$, $lag_{\chi }$ and $\varphi_{\chi }$ at AM frequencies 0.05 to 0.1 Hz are calculated for subsurface defects up till 5 mm deep. The 5 bit Barker coded VTWR is also compared with a 5 cycles LVT of the same AM frequency in terms of $Peak_{\chi }$ and $\varphi_{\chi }$ which correspond to the magnitude and phase of classical lock-in thermography. The results are presented in Figure 2.

Comparison of the $Peak_{\chi }$ values (see Figure 2a, the left axis) indicates that a lower AM frequency increases the magnitude of the peak value over the whole depth range. Although the magnitude of $Peak_{\chi }$ reduces by the depth, its ratio from VTWR to that of LVT (see Figure 2a, the right axis) rises significantly and at a higher rate for the higher AM frequency. Hence, this amplitude magnification effect of VTWR, which is gained through the broadband nature of the 5 bit Barker code, increases by the depth of the subsurface heat source and as such enhances the detectability of deeper defects in vibrothermography.

The left axis of Figure 2b shows that for both LVT and VTWR, the phase $\varphi_{\chi }$ increases by depth, but it reduces by lowering the AM frequency. Moreover, the broadband modulation introduced by VTWR leads to a lower phase $\varphi_{\chi }$ compared to LVT. This is explicitly shown in the right axis of Figure 2b which indicates that the degradation of $\varphi_{\chi }$ through VTWR is more pronounced at the lower AM frequency and for deeper defects.

Figure 2c further shows that the $lag_{\chi }$ of VTWR increases by the depth and also by lowering the AM frequency. The results explicitly show that although reducing the AM frequency from 0.1 to 0.05 Hz doubles the time period of the modulation cycle, the corresponding lag time $lag_{\chi }$ slightly changes and does not increase proportionally. This explains why lowering the AM frequency results in a lower phase delay at a particular depth (Figure 2b).

Overall, the results of analytical simulation demonstrate that the proposed VTWR technique outperforms LVT in terms of $Peak_{\chi }$ leading to an increased thermal signature particularly from deep defects. Moreover, it enables calculation of $lag_{\chi }$ as a measure for the depth of defect.

## 3. Experimental Validation of VTWR

**Figure 3:**
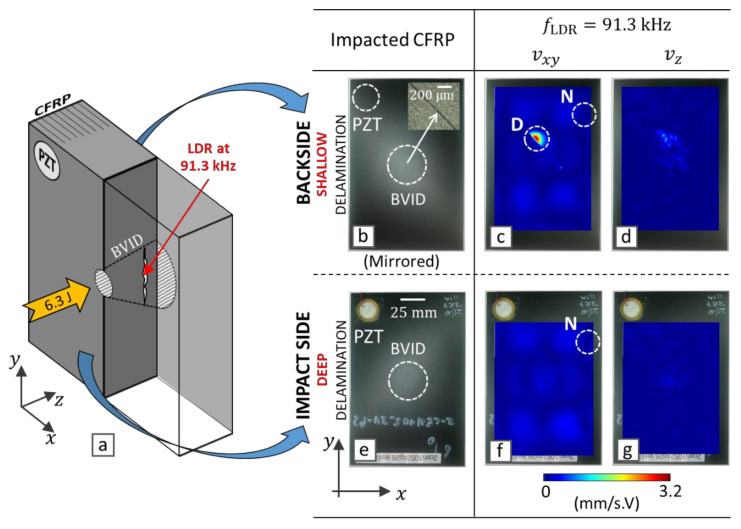
Impacted CFRP coupon of thickness 5.5 mm and corresponding vibrational response (in-plane $v_{xy}$, and out-of-plane $v_{z}$ ) measured by 3D infrared scanning laser Doppler vibrometer at a selected LDR frequency $f_{\mathrm{LDR}}=91.3\;\mathrm{kHz}$, (**a**) schematic presentation of the impacted CFRP, (**b**–**d**) backside results, (**e**–**g**) impact side results. D is the resonated fraction of barely visible impact damage (BVID), and N is an in-plane vibrational node of the CFRP coupon.

In this section, the enhanced depth resolvability of VTWR is further validated by experiment. For this purpose, an impacted CFRP coupon (Honda R&D, Wako, Saitama, Japan) with a quasi-isotropic lay-up ${\left[{\left(+45/0/-45/90\right)}_{3}\right]}_{S}$ and dimensions $100\times 150\times 5.5\;{\mathrm{mm}}^{3}$ was inspected (see Figure 3). The vibrational response of the sample was first measured by scanning laser Doppler vibrometry, and several local defect resonances were identified. An LDR of a deep backside delamination was selected to evaluate the performance of LVT and VTWR at various AM frequencies.

### 3.1. Experimental Set-Up and LDR Selection

The CFRP sample was impacted with a 7.1 kg drop-weight from a height of 0.1 m according to ASTM D7136 [53] resulting in an impact energy of 6.3 J, which introduced BVID including a hair-like surface crack at the backside (see the inset of Figure 3b). In order to induce broadband vibrations, a low-power PZT wafer (type EPZ-20MS64W from Ekulit, Ostfildern, Germany, with a diameter of 12 mm) was attached to the impact side of the CFRP coupon using Phenyl salicylate (Alfa Aesar by Thermo Fisher GmbH, Kandel, Germany). A Tektronix AFG-3021B arbitrary wave generator (Tektronix, Beaverton, OR, USA) together with a Falco System WMA-300 voltage amplifier (Falco Systems BV, TH Katwijk aan Zee, Netherlands) was used to supply a 150 Vpp sine sweep from 1 to 250 kHz to the PZT. The mechanical power transmitted to the sample is calculated to be about 200 mW according to [17] and, as such, confirms the low-power vibrational levels used in the here-described vibrothermography experiments.

In order to study the LDR behavior of the CFRP with BVID, the vibrational response of the sample was measured using a 3D infrared scanning laser Doppler vibrometer (PSV-500-3D XTRA, Polytec GmbH, Waldbronn, Germany) at a sampling frequency of 625 MS/s. The total measurement time of the laser Doppler vibrometry measurement was around 18 min for a scanning grid of 3166 points. BVID is comprised of a complex combination of damage features through the depth, introducing multiple LDRs measured at both the impact side and the backside of the sample [20]. Among the different measured LDRs, an LDR frequency of 91.3 kHz is chosen to be tested by vibrothermography which corresponds to a deep backside delamination in BVID (see Figure 3a). At this frequency, only the backside of the sample manifests a prominent in-plane LDR (see the indicated region D on Figure 3c). The (practically accessible) impact side (Figure 3f,g) is totally transparent to this LDR and merely indicates a global in-plane resonance of the sample. Whereas the sample is also experiencing a global in-plane resonance at this LDR frequency, it is expected that the vibrational nodes (e.g., the indicated region N on Figure 3c,f), which experience high in-plane strain energy density, heat up due to corresponding high damping losses. It should be noted that all surface maps of Figure 3 are shown with the same colormap scale so that the backside LDR behavior of the BVID at the frequency of 91.3 kHz is distinctively shown. Further, the vibrational response of the backside is mirrored so that the relative location of defects can be conveniently compared.

For vibrothermography measurements, the CFRP sample was inspected from both impact side and backside, and the depth resolvability in detecting the backside LDR was evaluated. Vibrational excitation was applied at the selected LDR frequency of 91.3 kHz, and was modulated at different AM frequencies 0.1, 0.075 and 0.05 Hz. VTWR is performed by using a 5 bit Barker coded waveform (see Figure 1a) and is compared with LVT of the same duration and energy (i.e., 5 cycles of sinusoidal excitation at the same AM frequency). The surface temperature was measured by a FLIR A6750sc infrared camera (FLIR, Wilsonville, OR, USA) at a sampling rate of 25 Hz. The camera has a cryo-cooled InSb detector, a pixel density of 640×512, a noise equivalent differential temperature (NEDT) of <20 mK, a bit depth of 14 bit and is controlled by edevis GmbH hardware–software (DisplayImg 6, edevis GmbH, Stuttgart, Germany). The output of the infrared camera is given in digital level (DL) scale, which corresponds to the intensity of the emitted infrared radiation. The measured thermal images were exported and further analyzed in MATLAB (R2020a, MathWorks, Natick, MA, USA).

### 3.2. VTWR versus LVT at the Selected LDR Frequency

**Figure 4:**
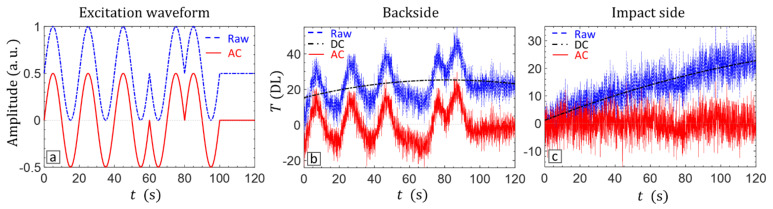
(**a**) A 5 bit Barker coded waveform extended with one bit of step heating. Raw thermal response and extracted alternating (AC) and direct (DC) components at a random pixel in the defected area: (**b**) backside and (**c**) impact side.

Initially, the CFRP sample was inspected at the AM frequency of 0.05 Hz. The 5 bit Barker coded waveform is extended with one bit of step heating as shown in Figure 4a. In this way, the latency of the thermal response to the coded waveform is taken into account and the corresponding AC component is properly decoupled [51]. The AC component is decoupled by filtering the DC component as a quadratic polynomial fit of the measured response. The raw temperature and the extracted AC component are shown in Figure 4 for a random pixel in the defected area at (b) the backside (shallow delamination) and (c) the impact side (deep delamination).

**Figure 5:**
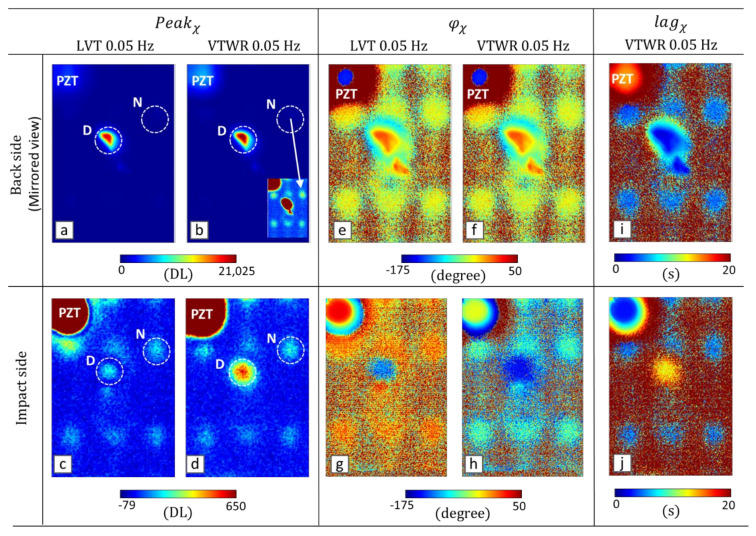
Vibrothermographic inspection of the CFRP sample at LDR frequency 91.3 kHz and AM frequency 0.05 Hz through LVT and VTWR from backside (top row) and impact side (bottom row): (**a**–**d**) $Peak_{\chi }$ (**e**–**h**) $\varphi_{\chi }$ and (**i**–**j**) $lag_{\chi }$. Note that the area heated by the PZT wafer is saturated in (**c**,**d**).

The surface maps of $Peak_{\chi }$, $\varphi_{\chi }$ and $lag_{\chi }$ at the AM frequency of 0.05 Hz are shown in Figure 5. The top and the bottom rows correspond to the inspection of the CFRP sample from the backside and the impact side, respectively. Due to the relatively low SNR of the thermal signal measured on the impact side (Figure 4c), a median filter with kernel size 3 × 3 pixels is applied to the $Peak_{\chi }$ maps (Figure 5d) for enhanced visualization of the heat sources. The results of both LVT and VTWR are shown with the same color scale for consistency, and the color bars of $Peak_{\chi }$ indicate the contrast with the sound area.

From the vibrational inspection (see Figure 3), it is clear that the chosen in-plane LDR frequency of 91.3 kHz activates a very deep fraction of the BVID in the backside. For the vibrothermographic inspection of the sample from the backside (see Figure 5a,b), the defect is clearly detected with a relatively high peak value, and there is no difference observable between the peak values obtained from LVT and VTWR. By saturating the colormap scale, a few other heating regions (e.g., the region N) are also detected, which correspond to the damping losses of the global in-plane vibrational nodes (see the inset of Figure 5b).

For the inspection from the impact side (see Figure 5c,d), the same vibrational nodes as well as the very deep defected area D (which was actually transparent to laser vibrometry from the impact side) are detected. Moreover, the $Peak_{\chi }$ at defect D is significantly higher for VTWR compared to LVT. Hence, the defect region D can be discerned from the vibrational node N in the VTWR results. The self-heating of the PZT wafer (attached on the impact side) leads to considerable heat generation, and is, therefore, saturated to improve the readability of the surface maps.

In terms of $\varphi_{\chi }$ (Figure 5e–h), the results of both LVT and VTWR provide a clear indication of the vibrational nodes and the defect region D. The results of the two techniques are comparable for the backside inspection. For the impact side however, they are significantly different. The phase map of LVT shows a higher dynamic range in which the phase of shallowest heat source (i.e., PZT) is close to the highest phase value of the range, and the phase of deepest heat source (defect region D) is close to the lowest phase value of the range. This is in good agreement with the results of simulation which showed outperformance of LVT in terms of $\varphi_{\chi }$.

In terms of $lag_{\chi }$ (Figure 5i,j), VTWR provides a meaningful indication of the defected area D and the vibrational nodes. For the backside surface map, the PZT area has the highest $lag_{\chi }$ indicating that this is the deepest heat source, while the defected area has the lowest $lag_{\chi }$ indicating that it is the shallowest heat source (and vice versa for the surface map of the impact side). Note that the upper limit of the colormap scale for $lag_{\chi }$ is set to 20 s to saturate noise and provide better indication of detected features.

**Figure 6:**
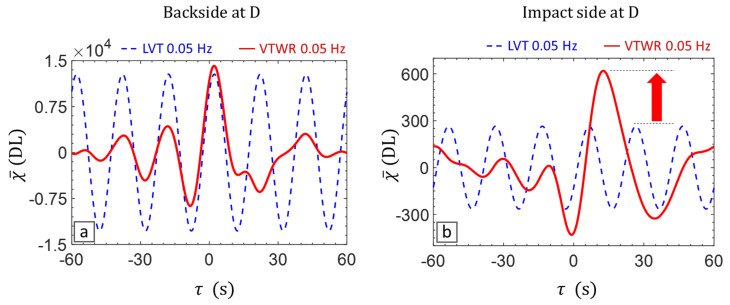
Compressed pulses of LVT and VTWR at AM frequency 0.05 Hz for the defected area D: (**a**) backside (shallow delamination) and (**b**) impact side (deep delamination).

The amplitude amplification efficiency of VTWR for inspection of deep defects is further demonstrated in Figure 6, through the cross-correlation curves of LVT and VTWR averaged over the defected area D. Application of VTWR on the backside (shallow delamination) results in a compressed pulse with a prominent peak, which is slightly higher than the amplitude of the sinusoid resultant from LVT. More importantly, application of VTWR on the impact side (deep delamination) results again in a compressed pulse, but now with a significantly higher amplitude compared to LVT.

### 3.3. VTWR versus LVT at Different AM Frequencies

**Figure 7:**
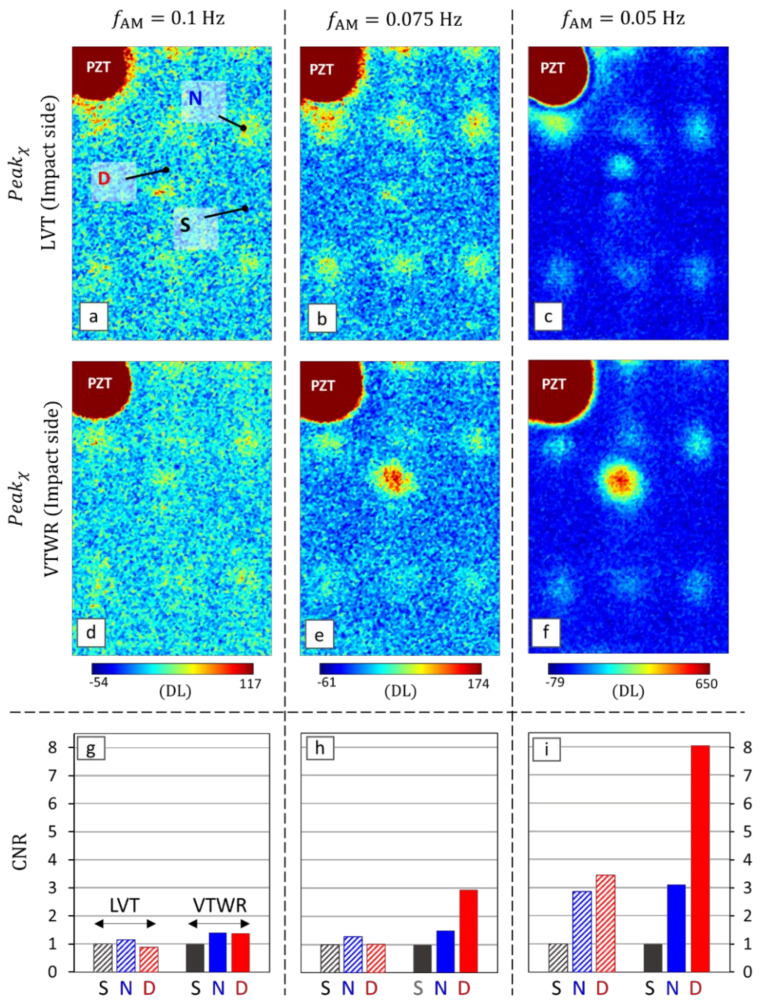
Inspection of the impact side of the CFRP sample at various AM frequencies: (**a**,**d**,**g**) 0.1 kHz, (**b**,**e**,**h**) 0.075 kHz and (**c**,**f**,**i**) 0.05 kHz. The top and the middle rows present the surface maps of $Peak_{\chi }$ resulting from LVT and VTWR, and the bottom row displays the CNR values of regions S, N and D calculated for both techniques. The color bars indicate the contrast with the sound area S.

For better understanding the efficiency of VTWR for the detection of deep defects in terms of the peak value $Peak_{\chi }$, the impact side of the sample was inspected at three different AM frequencies: 0.1, 0.075 and 0.05 Hz. The $Peak_{\chi }$ maps are shown for both LVT and VTWR in Figure 7a–f. Further, a contrast-to-noise ratio (CNR) is calculated for the defected area D and the vibrational node N, using the following equation [54]:

(12) $$\mathrm{CNR}=\frac{\left|{\bar{Peak}}_{\chi R}-{\bar{Peak}}_{\chi S}\right|}{\sigma_{S}}$$

where ${\bar{Peak}}_{\chi R}$ and ${\bar{Peak}}_{\chi S}$ are the average values of $Peak_{\chi }$ over an area of interest (i.e., defect D and node N) and the reference sound area S, respectively. $\sigma_{S}$ is the standard deviation of $Peak_{\chi }$ over the reference sound area S. The areas are indicated on Figure 7a.

With this definition, a CNR value < 1 indicates that the area of interest cannot be discerned from noise. The CNR values for both VTWR and LVT are given in Figure 7g–i and listed in Table 1.

The $Peak_{\chi }$ maps together with corresponding CNR values confirm the higher magnification efficiency of VTWR for the deep defects. At the highest AM frequency of 0.1 Hz, there is a minor indication of the vibrational nodes and the defected region D (Figure 7d). Upon lowering the AM frequency to 0.075 Hz (Figure 7e) and further to 0.05 Hz (Figure 7f), the defected region D is detected with a considerably higher magnitude compared to the vibrational nodes. At all AM frequencies, application of VTWR leads to an increased CNR value at the vibrational node N. However, more importantly, it increases the CNR of the defected region D with a higher rate. This leads to a distinct detectability of the (deep) backside defect D at the two lower AM frequencies 0.075 and 0.05 Hz. At these frequencies, VTWR allows for distinguishing the heating induced by the defected area D from the ‘misleading’ heating induced by a non-defected vibrational node like N. This is in clear contrast with the results obtained through LVT: even at the lowest AM frequency of 0.05 Hz, the CNR of the defect region D is at a similar level as the CNR of the vibrational node N.

## 4. Conclusions

Vibro-thermal wave radar (VTWR) technique was introduced as an efficient low-power non-destructive methodology for inspecting materials. The VTWR technique was benchmarked with the classical lock-in vibrothermography (LVT).

The VTWR was applied by binary phase modulation of the vibrational excitation using a 5 bit Barker coded waveform, and evaluating its cross-correlation with the resultant thermal response. Its performance was evaluated by means of a 1D analytical model in which the vibration-induced defect heating is simulated as subsurface heating sources. It was shown that the broadband nature of applied amplitude modulation in VTWR results in a magnification effect on the thermal signature, which is most pronounced for deep defects. It was further shown that LVT has a higher dynamic range in terms of phase. In turn, the pulse compression efficiency of VTWR provides a lag quantity which is a good measure for the defect depth.

Experimental validation was presented on an impacted CFRP coupon (impact energy 6.3 J) with thickness 5.5 mm. A backside delamination fraction was activated by vibrational excitation at the local defect resonance frequency of 91.3 kHz. Inspection of the impact side revealed that scanning laser Doppler vibrometry is unable to detect this very deep delamination. However, VTWR methodology is successful in detecting and resolving the very deep delamination in the impacted CFRP coupon and with a significantly higher contrast-to-noise ratio compared to LVT. Different AM frequencies were tested and the outperformance of VTWR was demonstrated.

Therefore, the proposed VTWR technique can be applied for full-field inspection of thick composite components and detection of very deep backside defects, using a noninvasive low-power vibrational excitation. Further analysis of the resultant lag quantity, enables characterization and depth estimation of defects.

## Figures and Tables

**Table 1:** CNR values of $Peak_{\chi }$ calculated for both LVT and VTWR techniques.

Inspection Technique	$f_{AM}=0.1\;\left(Hz\right)$			$f_{AM}=0.075\;\left(Hz\right)$			$f_{AM}=0.05\;\left(Hz\right)$		
	**S**	**N**	**D**	**S**	**N**	**D**	**S**	**N**	**D**
LVT	1	1.14	0.87	1	1.27	1.01	1	2.85	3.43
VTWR	1	1.40	1.38	1	1.49	2.94	1	3.10	8.04

## Data Availability

Data sharing not applicable.

## References

[1] Yang R., He Y. (2016). Optically and non-optically excited thermography for composites: A review. Infrared Phys. Technol..

[2] Ciampa F., Mahmoodi P., Pinto F., Meo M. (2018). Recent advances in active infrared thermography for non-destructive testing of aerospace components. Sensors.

[3] Poelman G., Hedayatrasa S., Segers J., Van Paepegem W., Kersemans M. (2020). An Experimental Study on the Defect Detectability of Time-and Frequency-Domain Analyses for Flash Thermography. Appl. Sci..

[4] Poelman G., Hedayatrasa S., Segers J., Van Paepegem W., Kersemans M. (2020). Adaptive spectral band integration in flash thermography: Enhanced defect detectability and quantification in composites. Compos. Part B Eng..

[5] Poelman G., Hedayatrasa S., Segers J., Van Paepegem W., Kersemans M. (2020). Multi-scale gapped smoothing algorithm for robust baseline-free damage detection in optical infrared thermography. NDT E Int..

[6] Netzelmann U., Müller D. (2020). Modified pulse-phase thermography algorithms for improved contrast-to-noise ratio from pulse-excited thermographic sequences. NDT E Int..

[7] Maierhofer C., Myrach P., Reischel M., Steinfurth H., Röllig M., Kunert M. (2014). Characterizing damage in CFRP structures using flash thermography in reflection and transmission configurations. Compos. Part B Eng..

[8] Reifsnider K., Henneke E.G., Stinchcomb W., Stinchcomb W.W., Duke J.C., Henneke E.G., Reifsnider K.L. (1980). The mechanics of vibrothermography. Mechanics of Nondestructive Testing.

[9] Rizi A.S., Hedayatrasa S., Maldague X., Vukhanh T. (2013). FEM modeling of ultrasonic vibrothermography of a damaged plate and qualitative study of heating mechanisms. Infrared Phys. Technol..

[10] Cavallone C., Colom M., Mendioroz A., Salazar A., Palumbo D., Galietti U. (2020). Sizing the length of surface breaking cracks using vibrothermography. NDT E Int..

[11] Chi X., Di Maio D., Lieven N.A.J. (2020). Health monitoring of bolted joints using modal-based vibrothermography. SN Appl. Sci..

[12] Guo X., Vavilov V. (2013). Crack detection in aluminum parts by using ultrasound-excited infrared thermography. Infrared Phys. Technol..

[13] Chi X., Di Maio D., Lieven N.A. (2019). Modal-based vibrothermography using feature extraction with application to composite materials. Struct. Health Monit..

[14] Holland S.D., Uhl C., Ouyang Z., Bantel T., Li M., Meeker W.Q., Lively J., Brasche L., Eisenmann D. (2011). Quantifying the vibrothermographic effect. NDT E Int..

[15] Katunin A., Wronkowicz-Katunin A., Wachla D. (2019). Impact damage assessment in polymer matrix composites using self-heating based vibrothermography. Compos. Struct..

[16] Solodov I., Busse G. (2013). Resonance ultrasonic thermography: Highly efficient contact and air-coupled remote modes. Appl. Phys. Lett..

[17] Solodov I., Rahammer M., Derusova D., Busse G. (2015). Highly-efficient and noncontact vibro-thermography via local defect resonance. Quant. InfraRed Thermogr. J..

[18] Fierro G.P., Ginzburg D., Ciampa F., Meo M. (2017). Nonlinear ultrasonic stimulated thermography for damage assessment in isotropic fatigued structures. J. Sound Vib..

[19] Dyrwal A., Meo M., Ciampa F. (2018). Nonlinear air-coupled thermosonics for fatigue micro-damage detection and localisation. NDT E Int..

[20] Segers J., Hedayatrasa S., Verboven E., Poelman G., Van Paepegem W., Kersemans M. (2019). In-plane local defect resonances for efficient vibrothermography of impacted carbon fiber-reinforced polymers (CFRP). NDT E Int..

[21] Hedayatrasa S., Segers J., Poelman G., Verboven E., Van Paepegem W., Kersemans M. (2020). Vibrothermographic spectroscopy with thermal latency compensation for effective identification of local defect resonance frequencies of a CFRP with BVID. NDT E Int..

[22] Wu D., Busse G. (1998). Lock-in thermography for nondestructive evaluation of materials. Rev. Générale Therm..

[23] Mandelis A., Thompson D.O., Chimenti D.E. (1987). Frequency modulated (FM) time delay-domain thermal wave techniques, instrumentation and detection: A review of the emerging state of the art in QNDE applications. Review of Progress in Quantitative Nondestructive Evaluation.

[24] Mulaveesala R., Vaddi J.S., Singh P. (2008). Pulse compression approach to infrared nondestructive characterization. Rev. Sci. Instrum..

[25] Tabatabaei N., Mandelis A. (2009). Thermal-wave radar: A novel subsurface imaging modality with extended depth-resolution dynamic range. Rev. Sci. Instrum..

[26] Tabatabaei N., Mandelis A. (2011). Thermal coherence tomography using match filter binary phase coded diffusion waves. Phys. Rev. Lett..

[27] Ghali V., Panda S., Mulaveesala R. (2011). Barker coded thermal wave imaging for defect detection in carbon fibre-reinforced plastics. Insight-Non-Destr. Test. Cond. Monit..

[28] Gong J., Liu J., Qin L., Wang Y.I. (2014). Investigation of carbon fiber reinforced polymer (CFRP) sheet with subsurface defects inspection using thermal-wave radar imaging (TWRI) based on the multi-transform technique. NDT E Int..

[29] Laureti S., Silipigni G., Senni L., Tomasello R., Burrascano P., Ricci M. (2018). Comparative study between linear and non-linear frequency-modulated pulse-compression thermography. Appl. Opt..

[30] Shi Q., Liu J., Wang Y., Liu W. (2018). Study on the Detection of CFRP Material with Subsurface Defects Using Barker-Coded Thermal Wave Imaging (BC-TWI) as a Nondestructive Inspection (NDI) Tool. Int. J. Thermophys..

[31] Shi Q., Liu J., Liu W., Wang F., Wang Y. (2019). Barker-coded Modulation Laser Thermography for CFRP Laminates Delamination Detection. Infrared Phys. Technol..

[32] Dua G., Arora V., Mulaveesala R. (2020). Defect Detection Capabilities of Pulse Compression based Infrared Non-destructive Testing and Evaluation. IEEE Sens. J..

[33] Rani A., Mulaveesala R., Kher V. (2021). An analytical approach for frequency modulated thermal wave imaging for testing and evaluation of glass fiber reinforced polymers. IOP SciNotes.

[34] Hedayatrasa S., Poelman G., Segers J., Van Paepegem W., Kersemans M. (2019). Performance of frequency and/or phase modulated excitation waveforms for optical infrared thermography of CFRPs through thermal wave radar: A simulation study. Compos. Struct..

[35] Hedayatrasa S., Poelman G., Segers J., Van Paepegem W., Kersemans M. (2019). Novel discrete frequency-phase modulated excitation waveform for enhanced depth resolvability of thermal wave radar. Mech. Syst. Signal Process..

[36] Hedayatrasa S., Poelman G., Segers J., Van Paepegem W., Kersemans M. (2021). On the application of an optimized Frequency-Phase Modulated waveform for enhanced infrared thermal wave radar imaging of composites. Opt. Lasers Eng..

[37] Yang R., He Y., Mandelis A., Wang N., Wu X., Huang S. (2018). Induction Infrared Thermography and Thermal-Wave-Radar Analysis for Imaging Inspection and Diagnosis of Blade Composites. IEEE Trans. Ind. Inform..

[38] Yi Q., Tian G.Y., Malekmohammadi H., Zhu J., Laureti S., Ricci M. (2019). New features for delamination depth evaluation in carbon fiber reinforced plastic materials using eddy current pulse-compression thermography. NDT E Int..

[39] Lu X., Yi Q., Tian G. (2020). A Comparison of Feature Extraction Techniques for Delamination of CFRP Using Eddy Current Pulse-Compression Thermography. IEEE Sens. J..

[40] Rantala J., Wu D., Busse G. (1996). Amplitude-modulated lock-in vibrothermography for NDE of polymers and composites. Res. Nondestruct. Eval..

[41] Dillenz A., Busse G., Wu D. (1999). Ultrasound lock-in thermography: Feasibilities and limitations. Diagnostic Imaging Technologies and Industrial Applications.

[42] Liu J., Gong J., Qin L., Wang H., Wang Y. (2014). Study of inspection on metal sheet with subsurface defects using linear frequency modulated ultrasound excitation thermal-wave imaging (LFM-UTWI). Infrared Phys. Technol..

[43] Renshaw J., Chen J.C., Holland S.D. (2011). The sources of heat generation in vibrothermography. NDT E Int..

[44] Vaddi J.S., Holland S.D. Identification of heat source distribution in vibrothermography. Proceedings of the AIP Conference.

[45] Truyaert K., Aleshin V., Van Den Abeele K., Delrue S. (2019). Theoretical calculation of the instantaneous friction-induced energy losses in arbitrarily excited axisymmetric mechanical contact systems. Int. J. Solids Struct..

[46] Rodriguez S., Meziane A., Pradère C. (2019). Thermal Chladni plate experiments to reveal and estimate spatially dependent vibrothermal source. Quant. InfraRed Thermogr. J..

[47] Vaddi J.S., Holland S.D., Kessler M.R. (2021). Loss modulus measurement of a viscoelastic polymer at acoustic and ultrasonic frequencies using vibrothermography. Measurement.

[48] Hahn D.W., Özisik M.N. (2012). Heat Conduction.

[49] Tabatabaei N. (2018). Matched-Filter Thermography. Appl. Sci..

[50] Mahafza B.R. (2016). Radar Systems Analysis and Design Using MATLAB Third Edition.

[51] Silipigni G., Burrascano P., Hutchins D.A., Laureti S., Petrucci R., Senni L., Torre L., Ricci M. (2017). Optimization of the pulse-compression technique applied to the infrared thermography nondestructive evaluation. NDT E Int..

[52] Maierhofer C., Röllig M., Gower M., Lodeiro M., Baker G., Monte C., Adibekyan A., Gutschwager B., Knazowicka L., Blahut A. (2018). Evaluation of Different Techniques of Active Thermography for Quantification of Artificial Defects in Fiber-Reinforced Composites Using Thermal and Phase Contrast Data Analysis. Int. J. Thermophys..

[53] ASTM International (2015). D7136/D7136M-15, A, Standard Test Method for Measuring the Damage Resistance of a Fiber-Reinforced Polymer Matrix Composite to a Drop-Weight Impact Event.

[54] Usamentiaga R., Ibarra-Castanedo C., Maldague X. (2018). More than fifty shades of grey: Quantitative characterization of defects and interpretation using snr and cnr. J. Nondestruct. Eval..

